# Supplementary material for: Functional and molecular heterogeneity of D2R neurons along dorsal ventral axis in the striatum
Source: Nat Commun. 2020 Apr 23;11:1957. doi: 10.1038/s41467-020-15716-9 (PMC7181842; doi:10.1038/s41467-020-15716-9)
Supplement: Supplementary file 1 — Supplementary Information [file 41467_2020_15716_MOESM1_ESM.pdf]

Supplementary information

Functional and molecular heterogeneity of D2R neurons along dorsal ventral axis in the striatum

Puighermanal et al.

Supplementary Figure 1

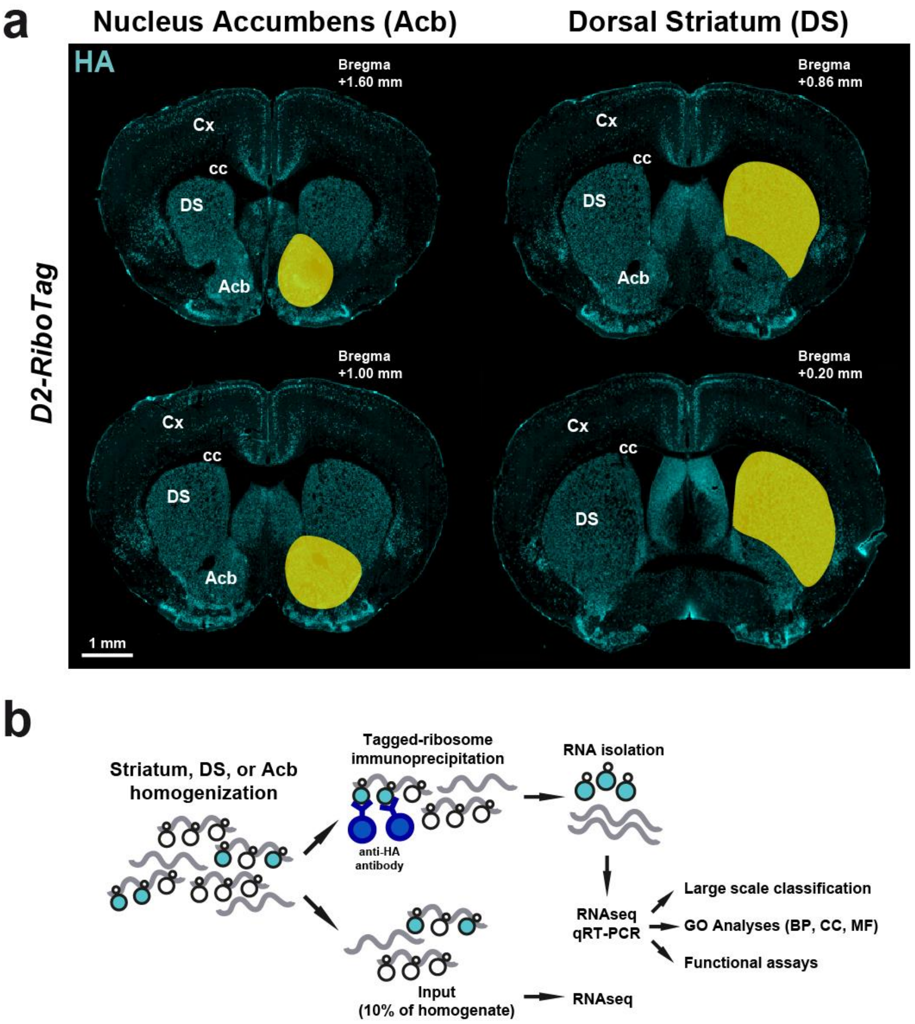

Supplementary Figure 1. Genetic strategy to isolate mRNAs selectively from D2R-expressing cells. (**a, b**) Dissection of DS and Acb in *D2-RiboTag* mice followed by RNAseq or qRT-PCR of the mRNAs bound to tagged ribosomes after HA-immunoprecipitation. DS: Dorsal Striatum, Cx: Cortex, Acb: Accumbens, cc: corpus callosum.

Supplementary Figure 2

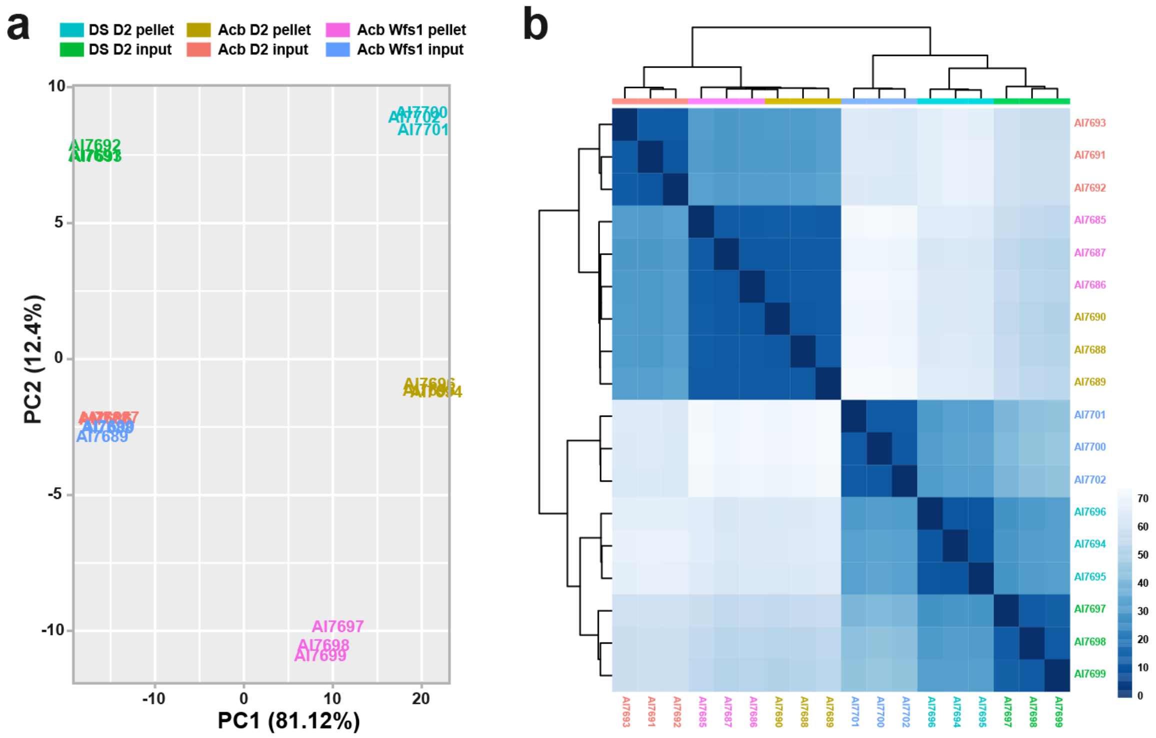

Supplementary Figure 2. RNAseq analysis reveals distinct translatome profiles between striatal D2R- and Wfs1-expressing cells as well as between DS and Acb. (**a**) Principal component analysis (PCA) of RNAseq gene expression of inputs and tagged-bound mRNAs from DS and Acb of *D2-RiboTag* and *Wfs1-RiboTag* mice. Each number corresponds to a sample of tissues from 3-4 mice. (**b**) Heatmap of a correlation matrix between the six experimental groups.

Supplementary Figure 3

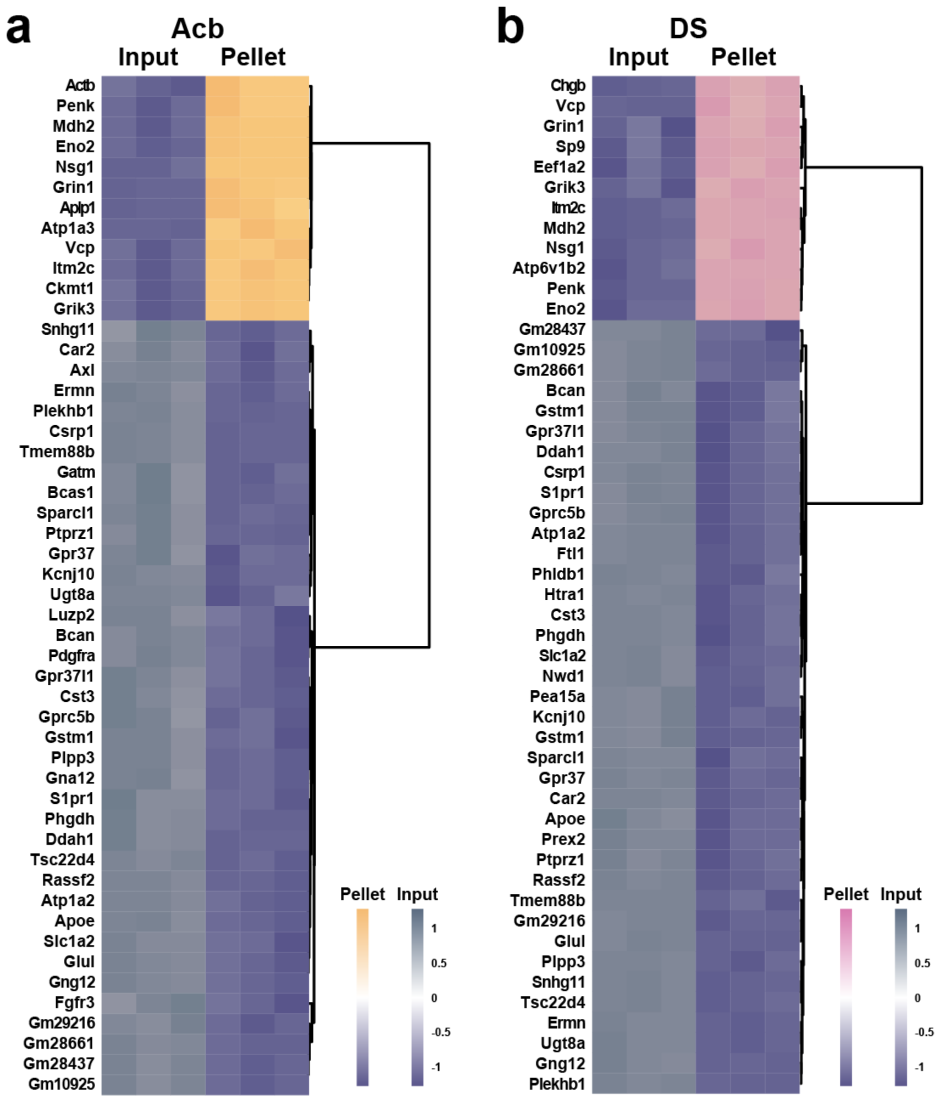

Supplementary Figure 3. Heatmaps of DS- and Acb-enriched genes from D2R neurons. (**a**,**b**) Heatmap of the top 50 genes most significantly enriched either in Acb (orange) or DS (pink) after the HA-immunoprecipitation from *D2-RiboTag* mice (pellet) compared to the input fraction.

Supplementary Figure 4

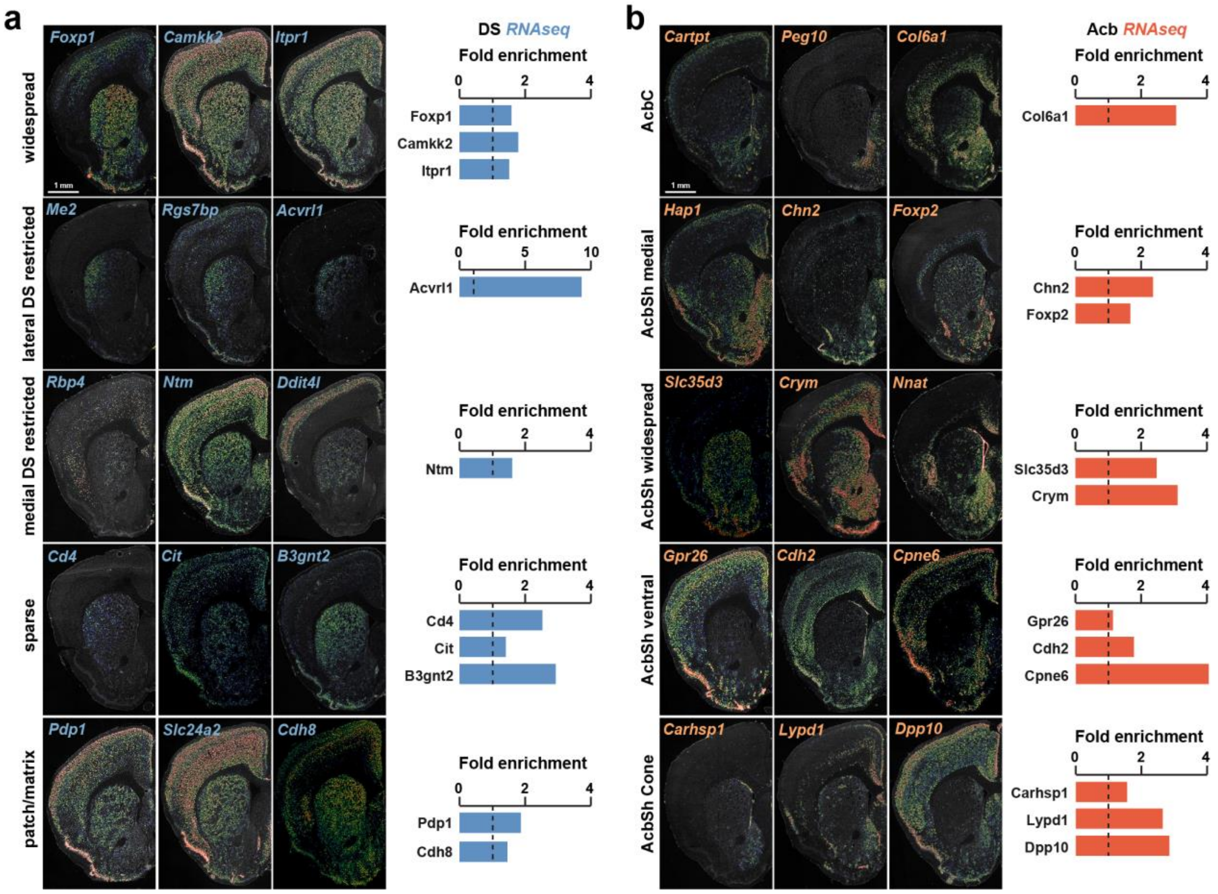

Supplementary Figure 4. ISH expression pattern of DS- and Acb-enriched genes. (**a**) Fold-change of DS-enriched genes found by RNAseq and corresponding ISH expression pattern from the *Allen Brain Atlas*. (**b**) Fold-change of Acb-enriched genes found by RNAseq and corresponding ISH expression pattern provided by the *Allen Brain Atlas*. Note the diversity of gene expression patterns allowing us to refine our classification taking into account distinct DS (lateral, medial, patch/matrix, widespread, sparse) and Acb (AcbC and medial, ventral, cone, and widespread AcbSh) subterritories.

Supplementary Figure 5

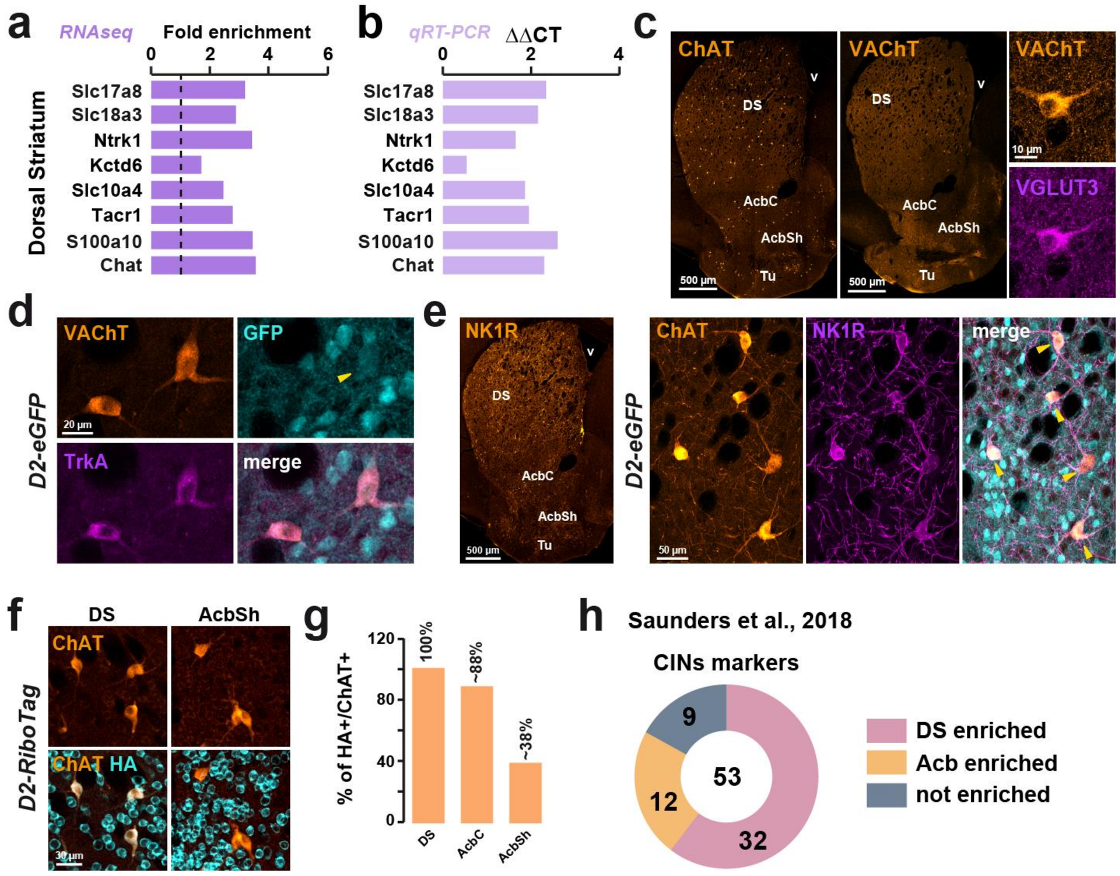

Supplementary Figure 5. Heterogeneity of cholinergic interneurons. (**a**, **b**) Fold-change and ΔΔCT of CIN-expressing genes enriched in the DS found by RNAseq (**a**) and confirmed by qRT-PCRs (**b**) in different *D2-RiboTag* mice. (**c**) Coronal striatal sections of VAChT and ChAT staining and double-staining of VAChT and VGLUT3. (**d**) Triple-immunolabeling for VAChT/TrkA/GFP in *D2-eGFP* mice. (**e**) Coronal striatal section of NK1R staining and triple-staining for NKR1 and ChAT/NK1R/GFP in *D2-eGFP* mice. (**f**) Double-staining of ChAT/HA in *D2-RiboTag* mice. (**g**) Percentage of ChAT/HA co-expressing cells in the DS, AcbC, and AcbSh. (**h**) Doughnut chart showing the overlap and distribution of D2R-enriched genes found in our study among the 53 CINs markers[1]. DS: Dorsal Striatum, AcbC: Accumbens Core, AcbSh: Accumbens Shell, Tu: olfactory tubercules, v: ventricule.

Supplementary Figure 6

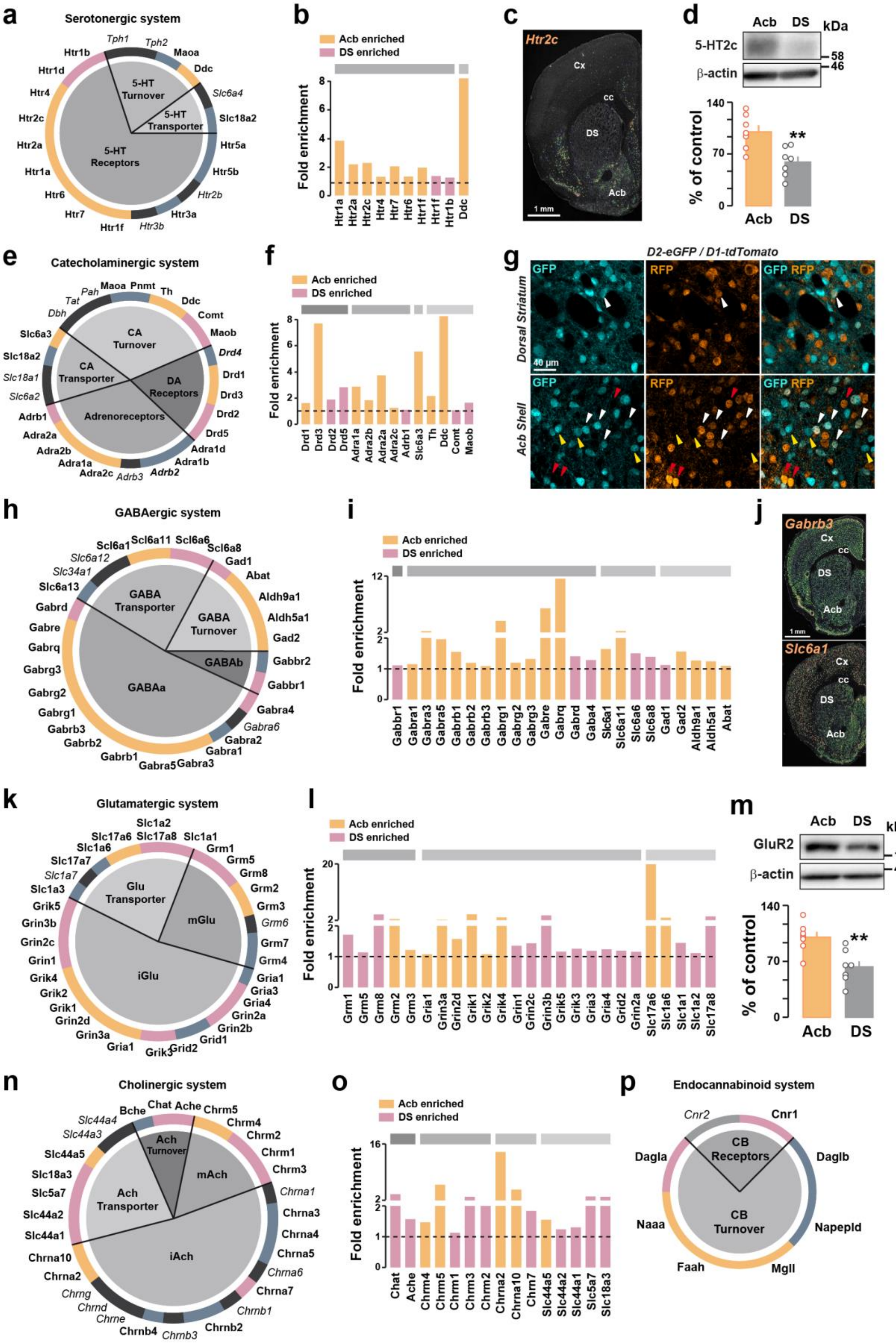

Supplementary Figure 6. Classification of D2R-enriched genes belonging to neurotransmitter systems according to their preferential expression in DS or Acb. (**a**) Classification of genes belonging to the serotonergic system. Genes are sorted according to receptors, transporters, or enzymes involved in 5-HT turnover and color-coded depending if they are enriched in Acb (orange), DS (pink), not differentially expressed between Acb and DS (gray), or not expressed (black). (**b**) Fold-change of statistically significant genes enriched in Acb and DS. (**c**, **d**) Htr2c as an example of an Acb-preferentially expressed gene illustrated by ISH from the *Allen Brain Atlas* (**c**) and at the protein level by WB (t_{12} = 3.523, p = 0.0042, two-sided *t* test, n = 7 mice/group) or IF (**d**). (**e**) Classification of genes belonging to the catecholaminergic system. (**f**) Fold-change of genes of the catecholaminergic system enriched in DS and Acb. (**g**) Double IF for GFP and RFP in *D2-eGFP/D1-tdTomato* double transgenic mice. D2R-, D1R-, and D2R/D1R-containing neurons are indicated with yellow, red, and white arrowheads respectively. (**h**) Classification of genes belonging to the GABAergic system. (**i**) Fold-change of genes of the GABAergic system enriched in DS and Acb. (**j**) ISH from the *Allen Brain Atlas* of the GABAergic system genes enriched in Acb. (**k**) Classification of genes belonging to the glutamatergic system. (**l**) Fold-change of genes of the glutamatergic system enriched in DS and Acb. (**m**) Enrichment of GluR2 in the Acb confirmed by WB analysis (t_{12} = 3.508, p = 0.0043, two-sided *t* test, n = 7 mice/group). (**n**) Classification of genes belonging to the cholinergic system. (**o**) Fold-change of genes of the cholinergic system enriched in DS and Acb. (**p**) Classification of genes belonging to the endocannabinoid system. All data from WB are presented as mean values ± SEM. DS: Dorsal Striatum, Cx: Cortex, Acb: Accumbens, cc: corpus callosum.

Supplementary Figure 7

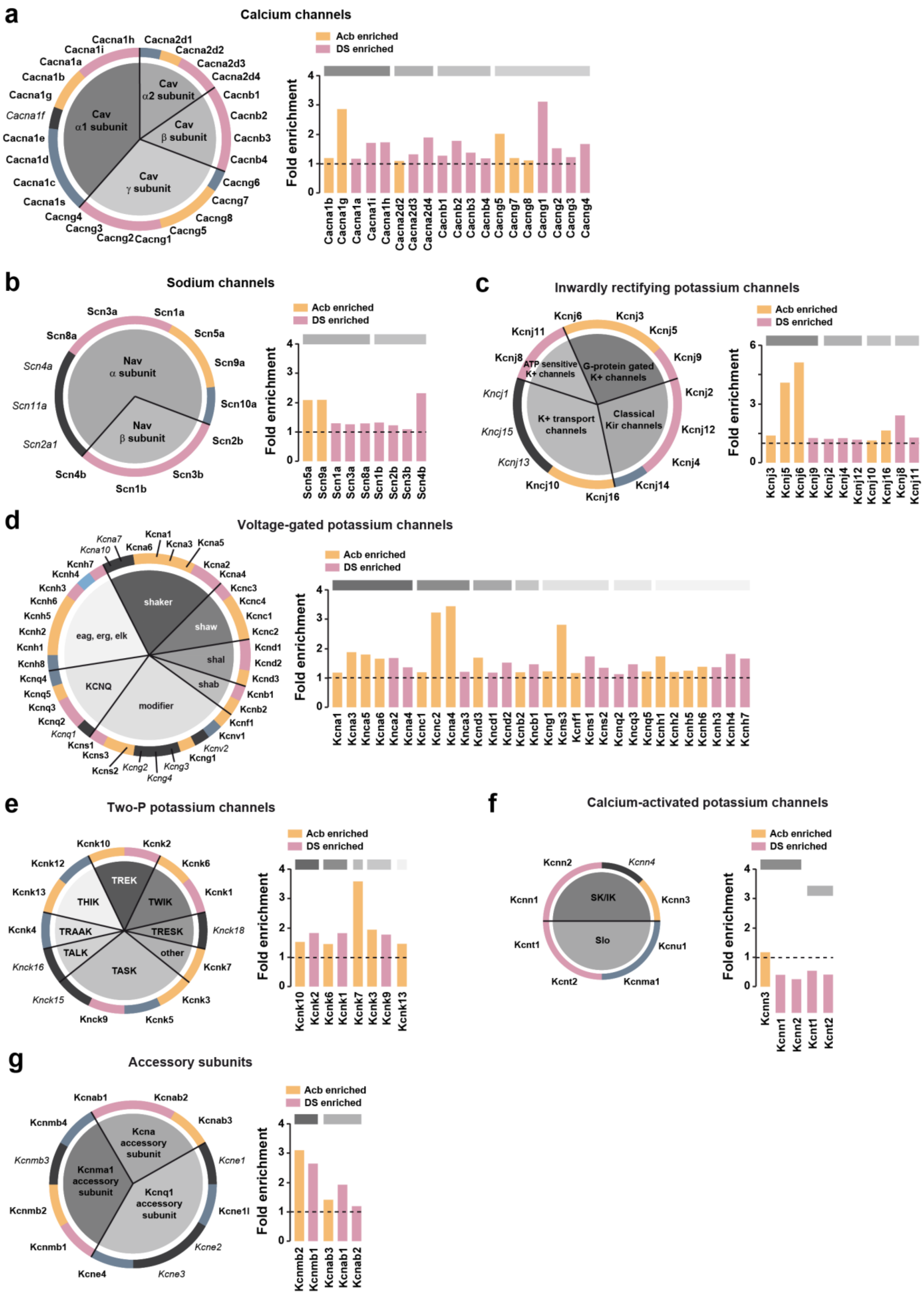

Supplementary Figure 7. Classification of D2R-enriched ion channels genes (IUPHAR/BPS database) according to their preferential expression in DS or Acb. (**a**) Classification of calcium channels-related genes and representation of the fold-enrichment in DS and Acb. (**b**) Classification of sodium channels-related genes and representation of the fold-enrichment in DS and Acb. (**c**) Classification of inwardly rectifying potassium channels genes and representation of the fold-enrichment in DS and Acb. (**d**) Classification of the distinct categories of voltage-gated potassium channels genes and representation of the fold-enrichment in DS and Acb. (**e**) Classification of the distinct categories of Two-P potassium channels genes and representation of the fold-enrichment in DS and Acb. (**f**) Classification of the calcium-activated potassium channels genes and representation of the fold-enrichment in DS and Acb. (**g**) Classification of the accessory subunits associated with potassium channels genes and representation of the fold-enrichment in DS and Acb.

Supplementary Figure 8

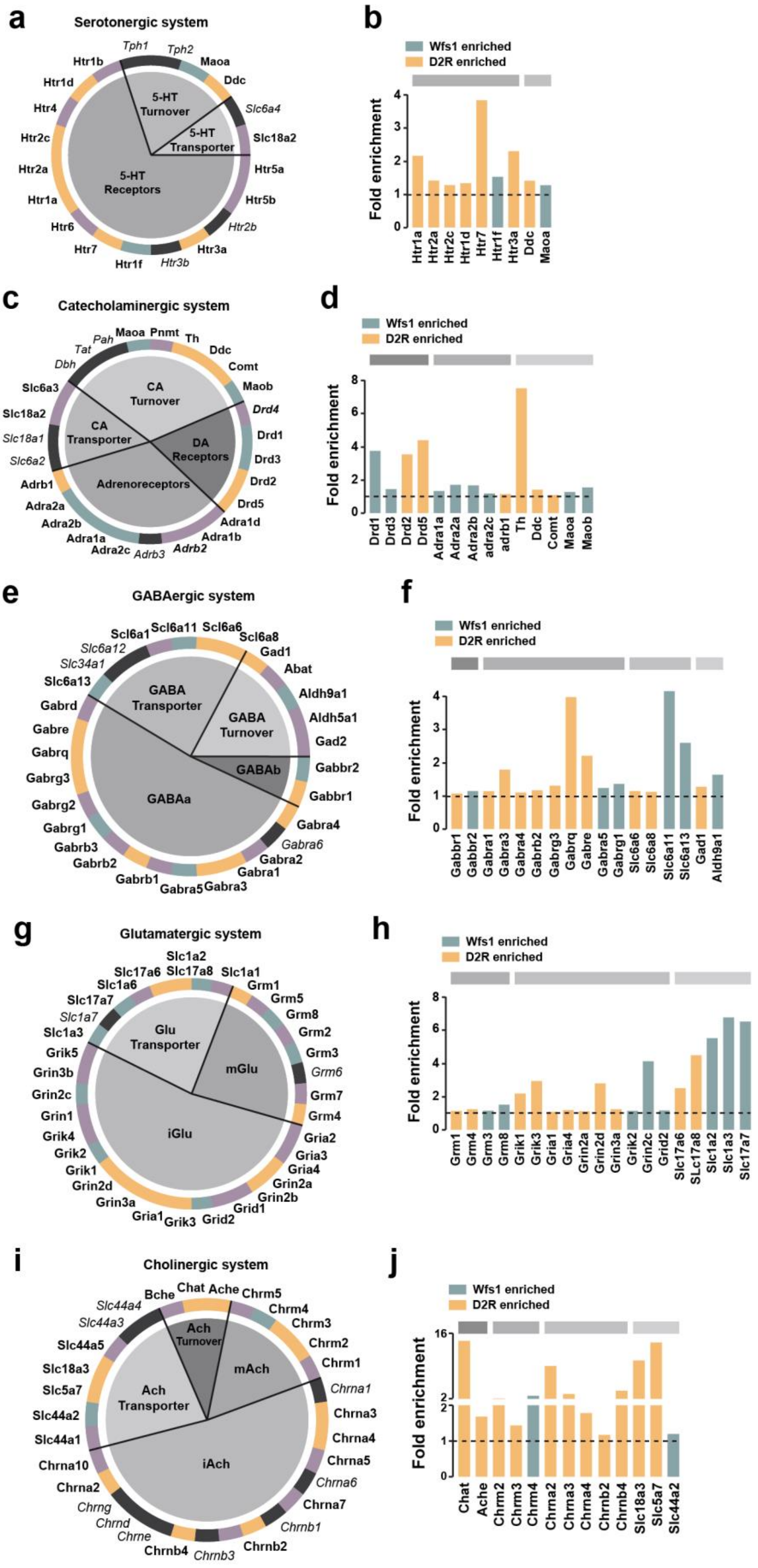

Supplementary Figure 8. Classification of Acb genes belonging to neurotransmitter systems according to their preferential expression in D2R cells or Wfs1 cells. (**a**) Classification of genes belonging to the serotonergic system. Genes are color-coded depending if they are enriched in D2R cells (orange), Wfs1 cells (green), not differentially expressed between Acb and DS (purple), or not expressed (black). (**b**) Fold-change of statistically significant genes enriched in D2R cells and Wfs1 cells. (**c**) Classification of genes belonging to the catecholaminergic system. (**d**) Fold-change of genes of the catecholaminergic system enriched in D2R cells and Wfs1 cells. (**e**) Classification of genes belonging to the GABAergic system. (**f**) Fold-change of genes of the GABAergic system enriched in D2R cells and Wfs1 cells. (**g**) Classification of genes belonging to the glutamatergic system. (**h**) Fold-change of genes of the glutamatergic system enriched in D2R cells and Wfs1 cells. (**i**) Classification of genes belonging to the cholinergic system. (**j**) Fold-change of genes of the cholinergic system enriched in D2R cells and Wfs1 cells.

Supplementary Figure 9

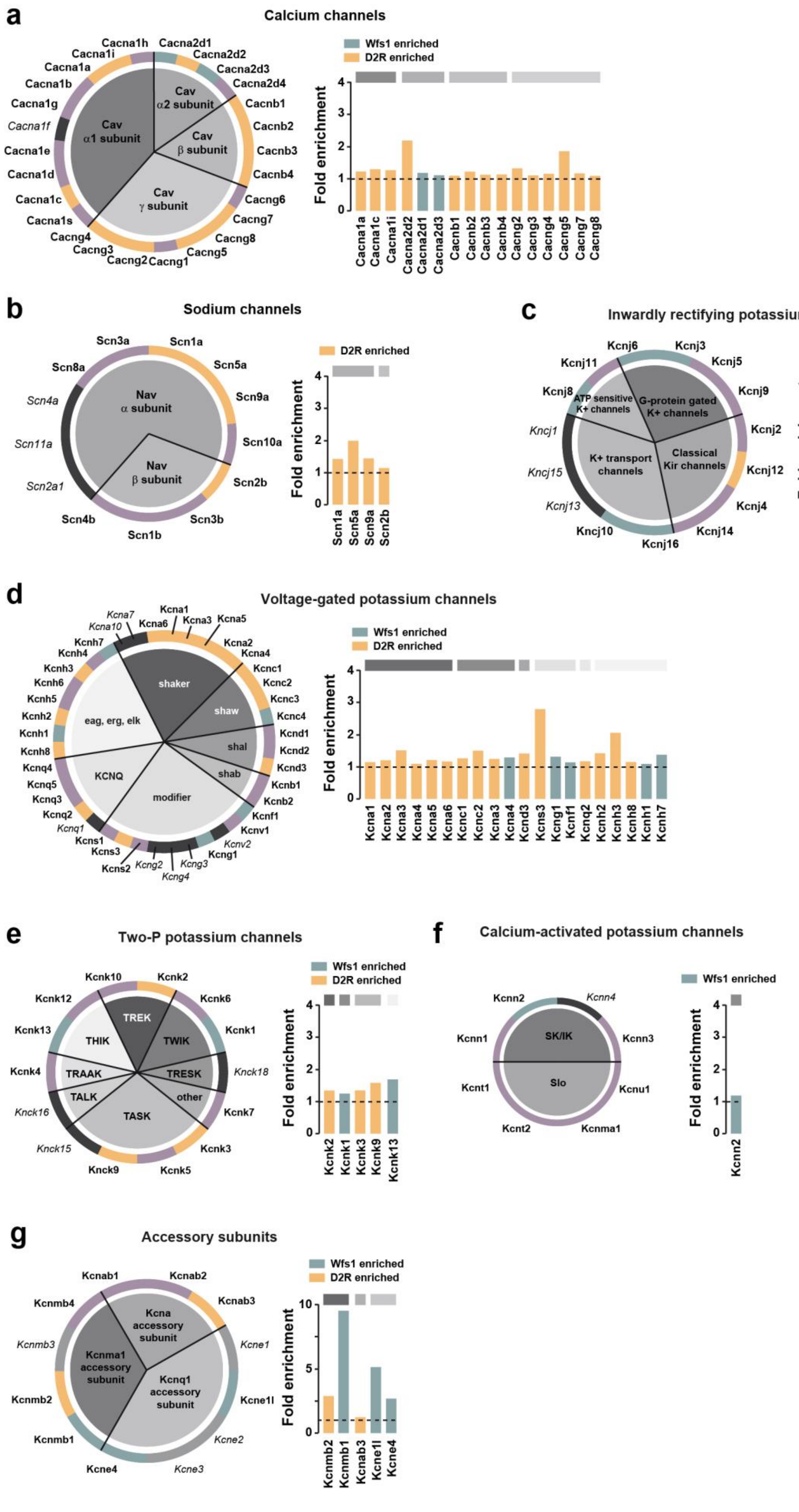

Supplementary Figure 9. Classification of Acb ion channels genes according to their preferential expression in D2R cells or Wfs1 cells. (**a**) Classification of calcium channels-related genes and representation of the fold-enrichment in D2R cells and Wfs1 cells. (**b**) Classification of sodium channels-related genes and representation of the fold-enrichment in D2R cells and Wfs1 cells. (**c**) Classification of inwardly rectifying potassium channels genes and representation of the fold-enrichment in D2R cells and Wfs1 cells. (**d**) Classification of the distinct categories of voltage-gated potassium channels genes and representation of the fold-enrichment in D2R cells and Wfs1 cells. (**e**) Classification of the distinct categories of Two-P potassium channels genes and representation of the fold-enrichment in D2R cells and Wfs1 cells. (**f**) Classification of the calcium-activated potassium channels genes and representation of the fold-enrichment in D2R cells and Wfs1 cells. (**g**) Classification of the accessory subunits associated with potassium channels genes and representation of the fold-enrichment in D2R cells and Wfs1 cells.

Supplementary Figure 10

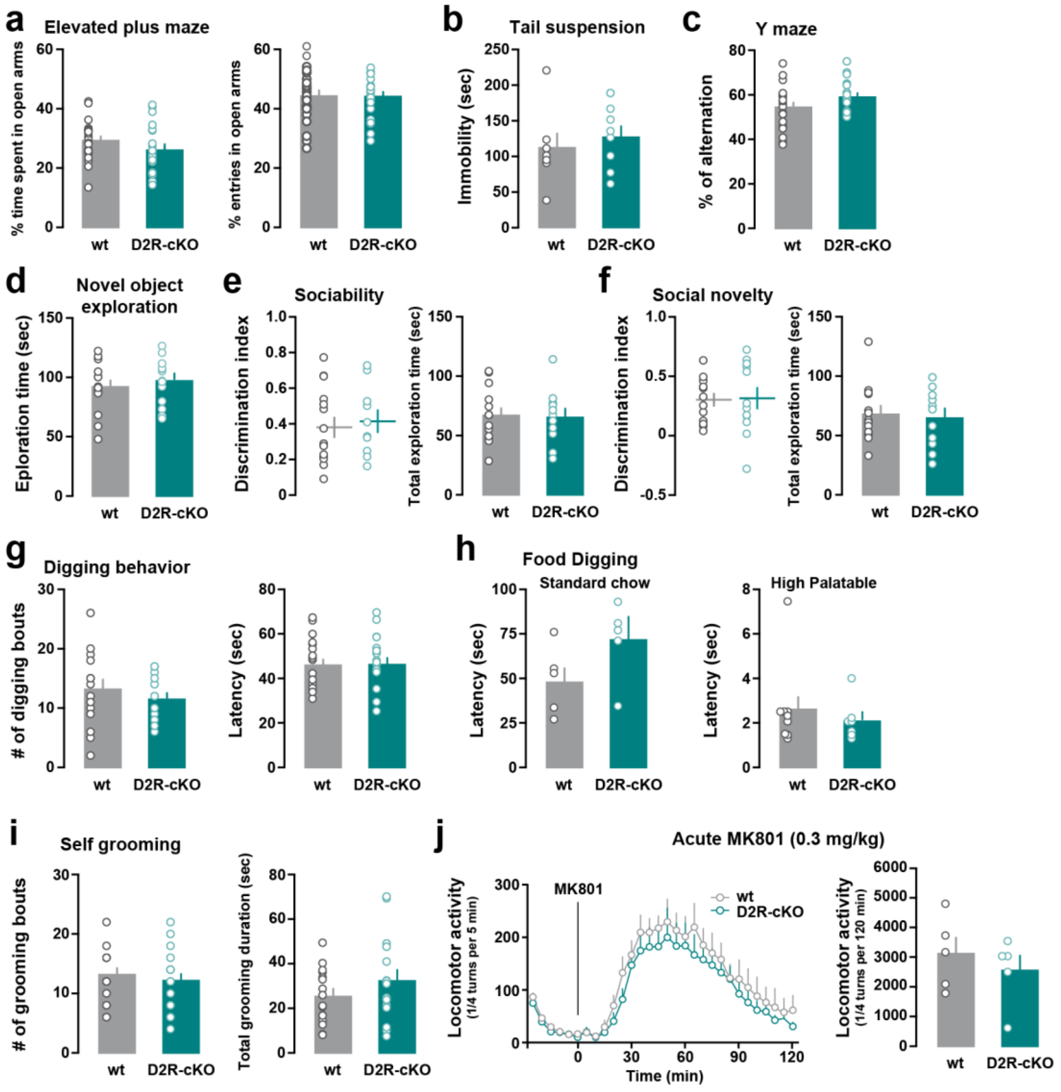

Supplementary Figure 10. Behavioral tests assessed in D2R-cKO mice. (**a**) Percentage of time spent (t_{44} = 1.537, p = 0.1315, two-sided *t* test, n = 22 wt and n = 24 D2R-cKO) and entries (t_{44} = 0.3361, p = 0.7384, two-sided *t* test, n = 22 wt and n = 24 D2R-cKO) in open arms during 5 min in the elevated plus maze. (**b**) Total immobility time over 6 min in the tail suspension test (t_{13} = 0.5741, p = 0.5757, two-sided *t* test, n = 7 wt and n = 8 D2R-cKO). (**c**) Percentage of alternations during 5 min in the Y-maze (t_{43} = 1.878, p = 0.0671, two-sided *t* test, n = 23 wt and n = 22 D2R-cKO). (**d**) Total exploration time over 30 min around a novel object placed in the center of an open field (t_{26} = 0.6543, p = 0.5186, two-sided *t* test, n = 14 mice/genotype). (**e**) Discrimination index of the time exploring Stranger#1 versus an empty wire cage (sociability)

(t_{23} = 0.4094, p = 0.6861, two-sided *t* test, n = 15 wt and n = 10 D2R-cKO) and total exploration time over 10 min (t_{23} = 0.1784, p = 0.86, two-sided *t* test, n = 15 wt and n = 10 D2R-cKO). (**f**) Discrimination index of the time exploring Stranger#2 versus Stranger#1 (social novelty) (t_{23} = 0.1263, p = 0.9006, two-sided *t* test, n = 14 wt and n = 11 D2R-cKO) and total exploration time over 10 min (t_{23} = 0.3289, p = 0.7452, two-sided *t* test, n = 14 wt and n = 11 D2R-cKO). (**g**) Total number of digging bouts over 3 min (t_{27} = 1.464, p = 0.1548, two-sided *t* test, n = 14 wt and n = 13 D2R-cKO) and latency to the first digging bout (t_{27} = 0.2425, p = 0.8102, two-sided *t* test, n = 14 wt and n = 13 D2R-cKO). (**h**) Goal-directed digging toward standard or palatable food, latency to the first digging bout to (standard: t_7 = 1.619, p = 0.1494, two-sided *t* test, n = 5 wt and n = 4 D2R-cKO; palatable: t_{16} = 0.3992, p = 0.7389, two-sided *t* test, n = 10 wt and n = 8 D2R-cKO). (**i**) Total self-grooming duration (t_{30} = 1.244, p = 0.2233) and number of grooming bouts over 10 min (t_{30} = 0.576, p = 0.5689, two-sided *t* test, n = 16 mice/genotype). (**j**) Horizontal activity over 30 min of habituation and over 120 min after MK801 administration (0.3 mg/kg) (Time: $F_{(29, 232)}$ = 25.90, $p < 0.0001$; Genotype: $F_{(1, 8)}$ = 0.6303, p = 0.4502; Interaction: $F_{(29, 232)}$ = 0.2703, $p > 0.9999$, two-way ANOVA repeated measures; t_8 = 0.7560, p = 0.4713, n = 5 mice/genotype). All data are presented as mean values ± SEM.

Supplementary Figure 11

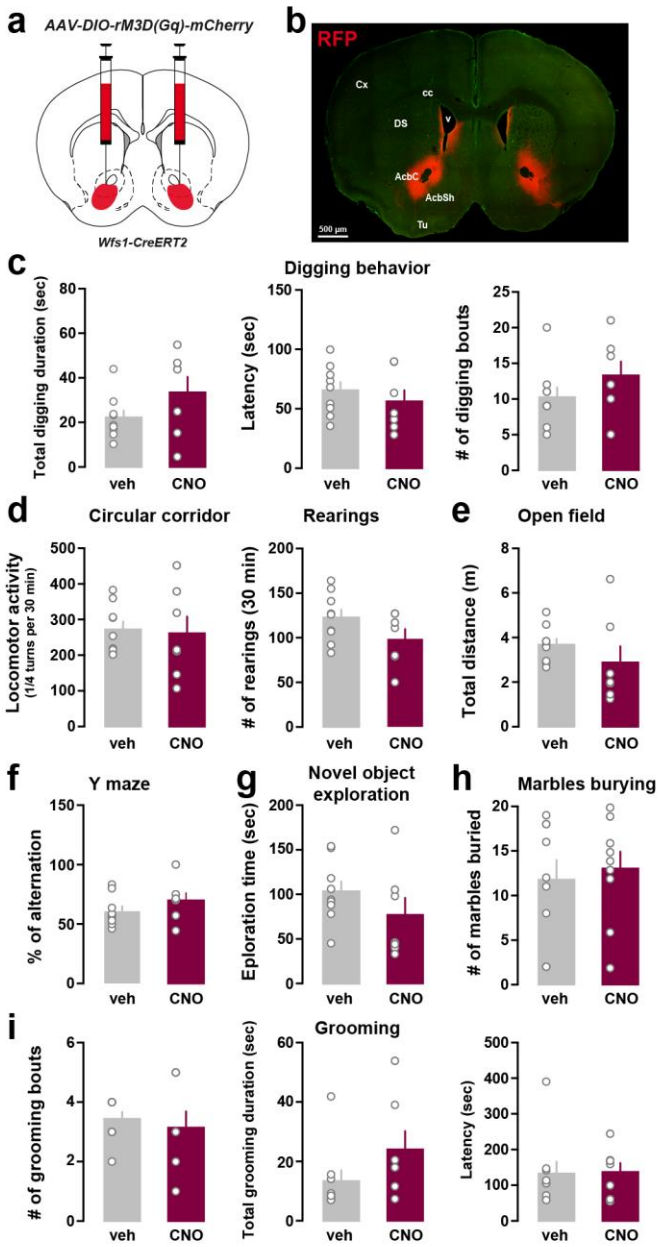

Supplementary Figure 11. Chemogenetic activation of Acb Wfs1 neurons. (**a**) Schematic of Cre-dependent AAV-hSyn-DIO-rM3D(Gq)-mCherry Acb injection in *Wfs1-CreERT2* mice. (**b**) Visualization of the mCherry expression (RFP) at the injection site. (**c**) Total duration (t_{14} = 1.569, p = 0.139, two-sided *t* test, n = 9 Veh- and n = 7 CNO-treated mice) and number of digging bouts over 3 min (t_{14} = 1.286, p = 0.2194, two-sided *t* test, n = 9 Veh- and n = 7 CNO-treated mice) and latency to the first digging bout (t_{14} = 0.8173, p = 0.4275, two-sided *t* test, n = 9 Veh- and n = 7 CNO-treated mice). (**d**) Horizontal (t_{14} = 0.2223, p = 0.8273, two-sided *t*

test, n = 9 Veh- and n = 7 CNO-treated mice) and vertical (t_{14} = 1.666, p = 0.118, two-sided *t* test, n = 9 Veh- and 7 CNO-treated mice) activity over 30 min in a circular corridor. (**e**) Total distance traveled over 30 min (t_{14} = 1.118, p = 0.2824, two-sided *t* test, n = 9 Veh- and n = 7 CNO-treated mice). (**f**) Percentage of alternations during 5 min in the Y-maze (t_{14} = 1.216, p = 0.2439, two-sided *t* test, n = 9 Veh- and n = 7 CNO-treated mice). (**g**) Total exploration time over 30 min around a novel object placed in the center of an open field (t_{14} = 1.22, p = 0.2425, two-sided *t* test, n = 9 Veh- and n = 7 CNO-treated mice). (**h**) Number of marbles buried after 20 min (t_{14} = 0.8754, p = 0.3962, two-sided *t* test, n = 9 Veh- and n = 7 CNO-treated mice). (**i**) Total number of grooming bouts over 10 min (t_{14} = 0.5418, p = 0.5965, two-sided *t* test, n = 9 Veh- and n = 7 CNO-treated mice), self-grooming duration (t_{14} = 1.551, p = 0.1431, two-sided *t* test, n = 9 Veh- and n = 7 CNO-treated mice) and latency to the first grooming bout (t_{14} = 0.09162, p = 0.9283, two-sided *t* test, n = 9 Veh- and n = 7 CNO-treated mice). All data are presented as mean values ± SEM. DS: Dorsal Striatum, Cx: Cortex, AcbC: Accumbens Core, AcbSh: Accumbens Shell, Tu: olfactory tubercules, v: ventricle, cc: corpus callosum.

Supplementary Figure 12

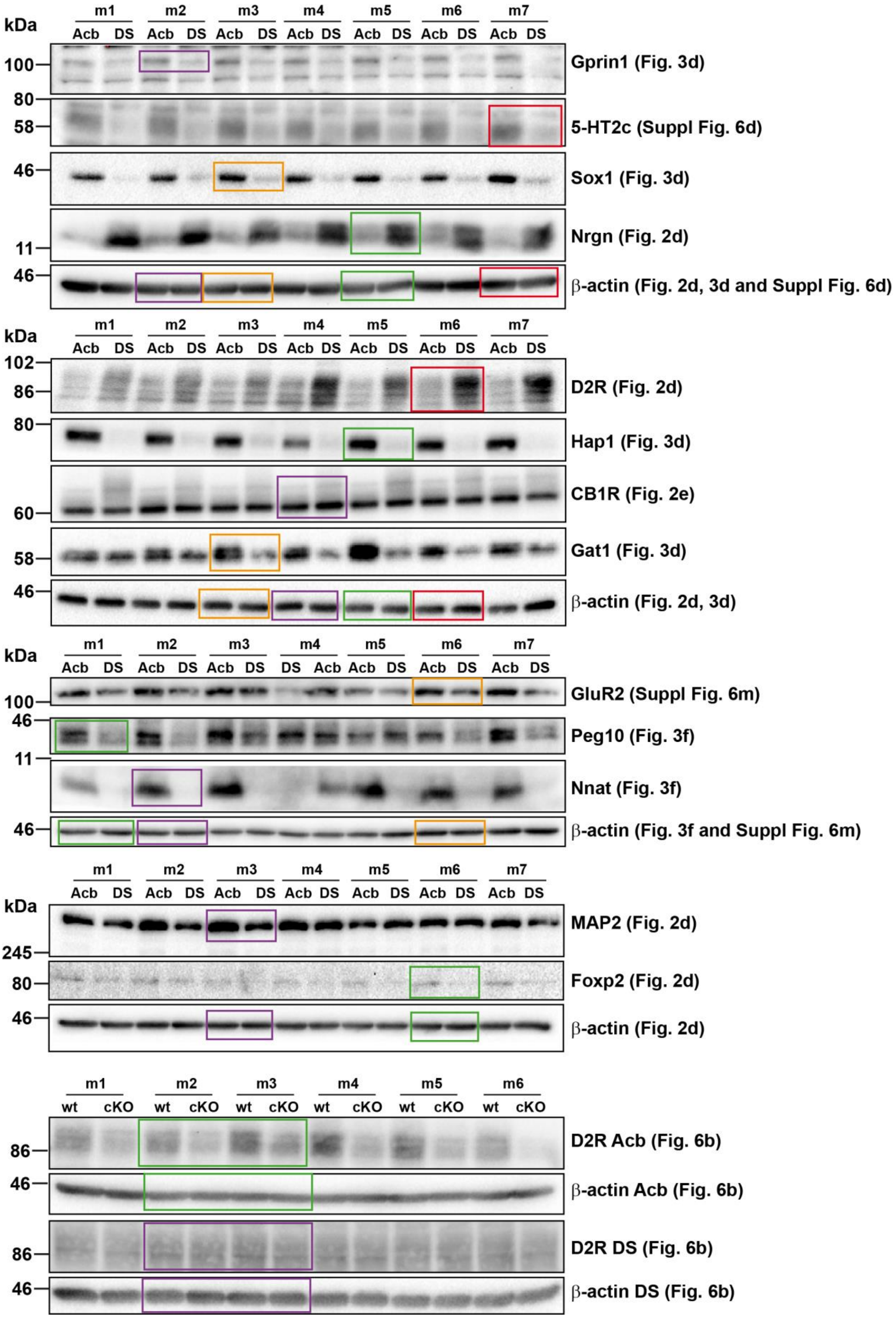

Supplementary Figure 12. Full immunoblots related to Fig. 2d, 2e, 3d, 3f, 6b and Supplementary Fig. 6d and 6m. Membranes were usually cut horizontally producing strips at different molecular weights to be probed in parallel with different primary antibodies. Colored boxes indicate bands shown in corresponding Figures. Molecular weight markers positions (kDa).

Supplementary Resources

Mouse lines used in the study

**Article's nomenclature** (Mouse line)	**Mouse strain**	**Citation**
D2-eGFP *(Drd2-eGFP)*	Tg(Drd2-EGFP)S118Gsat/Mmnc	2
D1-eGFP *(Drd1a-eGFP)*	Tg(Drd1-EGFP)X60Gsat/Mmmh	2
D2-eGFP/D1-tdTomato *(Drd2-eGFP/Drd1a-tdTomato)*	Tg(Drd2-EGFP)S118Gsat/Mmnc<br>B6.Cg-Tg(Drd1a-tdTomato)6Calak/J	2<br>3
Th-eGFP *(Th-eGFP)*	Tg(Th-EGFP)6-7Okn	4
D2-Cre *(Drd2-Cre)*	Tg(Drd2-cre)ER44Gsat/Mmucd	5
Wfs1-CreERT2 *(Wfs1-Tg3-CreERT2:Cre)*	B6.C3-Tg(Wfs1-cre/ERT2)3Aibs/J	6
RiboTag *(RiboTag-loxP/loxP)*	B6N.129-Rpl22tm1.1Psam/J	7
Drd2-floxed *(Drd2-loxP/loxP)*	B6.129S4(FVB)-$Drd2^{tm1.1Mrub}$/J	8
D2-RiboTag *(Drd2-Cre:RiboTag)*		9
Wfs1-RiboTag *(Wfs1-Tg3-CreERT2:Cre:RiboTag)*		10
D2R-cKO *(Wfs1-Tg3-CreERT2:Cre:Drd2-loxP/loxP)*		present study

List of Primary Antibodies

Antigen	Species	Dilution	Supplier/Catalog no./References)
HA	Mouse	1:1000 (IF)	Covance (#MMS-101R)
HA	Rabbit	1:1000 (IF)	Rockland (#600-401-384)
DARPP-32	Rabbit	1:1000 (IF)	Cell Signaling Technology (#2306)
Calretinin	Rabbit	1:1000 (IF)	Swant (#7699/3H)
Parvalbumin	Rabbit	1:1000 (IF)	Swant (#PV25)
NPY	Rabbit	1:500 (IF)	Abcam (#ab10980)
SOM	Rabbit	1:400 (IF)	Millipore (#AB5494)
Gat1	Rabbit	1:350 (IF); 1:1000 (WB)	Millipore (#AB1570)
Gprin1	Rabbit	1:500 (IF); 1:1000 (WB)	Proteintech Group (#13771-1-AP)
Hap1	Mouse	1:1000 (WB)	ThermoFischer Scientific (#MA1-46412)
Foxp2	Rabbit	1:500 (IF); 1:1000 (WB)	Abcam (#ab16046)
MAP2	Mouse	1:500 (IF); 1:2000 (WB)	Sigma-Aldrich (#M4403)
Sox1	Rabbit	1:1000 (WB)	Cell Signaling Technology (#4194)
Nrgn	Rabbit	1:500 (IF); 1:1000 (WB)	Santa-Cruz (#sc-50401)
D2R	Rabbit	1:500 (IF); 1:1000 (WB)	Frontier Institute (#D2R-Rb-Af960)
ChAT	Goat	1:500 (IF)	Millipore (#AB144)
GFP	Chicken	1:1000 (IF)	Life Technologies (#A10262)
NK1R	Rabbit	1:400 (IF)	Sigma-Aldrich (#S8305)
VAChT	Rabbit	1:500 (IF)	Synaptic System (#139103)
VGLUT3	Guinea Pig	1:500 (IF)	ref 11
TrkA	Rabbit	1:400 (IF)	Millipore (#06-574)
5-HT2c	Rabbit	1:500 (IF); 1:1000 (WB)	ref 12
CB1R	Rabbit	1:1000 (IF); 1:1000 (WB)	Frontier Institute (#CB1-Rb-Af960)
RFP	Rabbit	1:1000 (IF)	MBL (#PM005)
β-actin	Mouse	1:40000 (WB)	Abcam (#AB6276)
Dlk1	Mouse	1:400 (IF)	Adipogen (#AG-20A-0057)
Peg10	Rabbit	1:1000 (WB)	ref 13
Nnat	Rabbit	1:1000 (WB)	Abcam (#AB27266)
TH	Mouse	1:1000 (IF)	Millipore (#mab318)
GluR2	Mouse	1:1000 (WB)	Millipore (#MAB397)

Sequences of PCR primers

Cell-types	Genes	PCR primers *Forward*	*Reverse*
SPNs	*Pdyn*	AAGCCTGCCAGGGACAAAG	CCCCCACGCAGATCTCAA
	Drd1	TCGAACTGTATGGTGCCCTT	TGGGGTTCAGGGAGGAATTC
	Tac1	TGGACTAATGGGCAAAAGAGC	CGTTCACTGCTCACTGACAC
	Drd2	CTCTTTGGACTCAACAACACAGA	AAGGGCACGTAGAACGAGAC
	Adora2	CAGAGTTCCATCTTCAGCCTC	CACCCAGCAAATCGCAATG
	Penk1	CTACAGTGCAGGCGGAATGC	GTCCTTCACATTCCAGTGTGC
	Cnr1	CAGAGAGCCAGCCCCTTGG	AGGTGGTATCTGCAAGGCCG
	Trnp1	GTCATCTACGCGGAGGAGTCA	GGTATCCAGAGAAGGTGCGG
	Lpcat4	CACCTGTTGCTGGGTTCAC	GGTATCTGGGAGGTGCTTCG
	Kctd17	TCCACTATGTCCGACGGCTG	GAACTCGGCCTGGTCCTCAC
	Trpc3	GGTGGTCGTTTTACTCAACATGC	CCAGACTGAAGGGTGGAGGT
	Ace	GGGTCCCCTGCACAAGTGT	GATCAGCTTCATGGCCTCTGG
	Dab2ip	CAAGCAGCTGTAGATTCCAAACA	CTTTCAGCTGTGTGAGGGCA
	Me2	GCTTTACCCGTCGCTGGCTA	CCTTGTCCTCGGGTTCTGGG
	Rgs4	GACATGAAACATCGGCTGGG	GTTTTCCAACGATTCAGCCC
	Itga5	TGCAGTGGACCAAGGCAGAA	GCATCTGAGGTGGCTGGAGG
	Coch	TGATGATGTCCGAGGCCCTG	TCATCCAGCGGTGCCCAAG
	Tbc1d8	TGTGACCGACATTGCTGACCTG	TGGTGGTGTCCTCGTGTCCT
	Gpr155	AGCAGCAGACAGAGAATCCC	TTCGATGAGCCAGTTCACCA
	Rasd2	GCAACCACCCATTCCCTGCC	CCACAGATCACCATGGGCAGC
	Rgs7bp	TGCTGAAACACCTGCCCTGG	GGCATCGTTTCCCTGAGTTTGCT
	Slc24a2	CAGTGTCATAGTGGCCCGAAA	TGAAGAGAAGGACGATGGCAC
	Kcnk2	TCACTGGGACCTCGGAAGCT	GCTGATCACCAACCCCAGCC
	Ddit4l	GCAGTAAGAACCCGGCCAGC	CGTTGAGGTTGGGCTCAGGG
	Ccnd2	GATGCCCTGACAGAGCTGCT	GCTCTTGACGGAACTGCTGC
	Peg10	TGCTCTGATTACTGCCTGTATTCAC	CGTCACGAGCCAGCCTTCT
	Calcr	GCGTAGTTAGTGCTCCTC	CTTCGTTGTTGCTGATTGG
	Cbln4	TGTGGCACCGAGGAAAGGAA	CGGCTTCACGGGTCACATCT
	Amotl1	GCTCTGTCCCATCCATCGCT	ATGCCTGTCCCTGGTGTTCC
	Gabrg1	AAACCACCAGAGGCAGGAAG	AGGACCCAGTTCTGCAGTCA
	Ntn1	CTGGCAAGACCTGCAATCAA	CTGCTGGCTGCAGTGGTG
	Stard5	TGTGGACTTGGTGCTGGTGA	TCCTCTCACAAAACCGGGCT
	Lrrn3	TACTGGGGCAAGAAGACAGC	TCCCATGCTTGTTCAGACTTGC
	Myo16	ACCATGGGACTCAACTCTCCA	CCCCATGCCACTCAGACACA
	Nts	GCTGACCATCTTCCAGCTCC	CCCCTCTTGAGAATGTAGGGCC
	Cartpt	CGAAGCGTTGCAAGAAGTCCT	ACACAGCTTCCCGATCCTGG
	Nnat	GGCGTAGGCACCACATTCTG	GCTGTTCGATCTTCATGGTAGGAT
	Hap1	TGGGAAGAGTCATCGCCAGC	TCAGAGATGTTCGGAGCTCCTC
	Trhr	GAGCTAGAAGATATCACCGTCACCG	TGCCTGAAGACATCTGTTGCTG
	Hpcal4	ACTGGGCCTTTGAGATGTACGATC	CTTGAAGATCTTGTCCACACGCTG
	Gfra1	TGTATCGGGCAGTACACATCTCTG	CAATGATACAGACAACAGGGCAGC
	Dlk1	TGCACACCTGGGTTCTCTGG	CCTGGCCCTCATCATCCACG
	Fam126a	TGGAGAATGGGATCTAGCTCAG	GAGGAGGTTGGTGTGATCTCC
ChAT INs	*Slc17a8*	TGTCCCCTCATTGTTGGTGCA	TCCCCAGAAGCGAAGACCCC
	Slc18a3	GGCCTCGCTACCCCACAGAA	CCCAGGCCAATAAGCAGCGG
	Ntrk1	TTGGGGTGGTGCTCTGGGAG	AGACATCAGGAGGGCAGGCG
	Kctd6	TGGATAATGGGGACTGGGGCT	GTCTCGGGCTGTGGGGAAGT
	Slc10a4	GCAAGCATTCCCGCAACTGT	CACAGTCCGCTTGCAGTTGG
	Tacr1	CCCCTCATAATCACCAGCACTGA	CCTCCTGCCCTACATCAACCC
	S100a10	CCGAGATGGCAAAGTGGGCT	TGCTCACAAGAAGCAGTGGGG
	Chat	CCATTGTGAAGCGGTTTGGG	GCCAGGCGGTTGTTTAGATACA
PV INs	*Pvalb*	TCGATGACAGACGTGCTCAG	CTTCACCTCATCCGGGTTCT
TH INs	*Th*	CCGTGCAGCCCTACCAAGAT	CCGGATGGTGTGAGGACTGTC
CR INs	*Calb2*	TGAGAATGAACTGGACGCCCTC	GTAGAGCTTCCCTGCCTCGG
NPY INs	*Npy*	GCTCTGCGACACTACATCAA	GGCGTTTTCTGTGCTTTCCT
SOM INs	*Sst*	CTGTCCTGCCGTCTCCAGTG	CTCTGTCTGGTTGGGCTCGG
Glial cells			
Astrocytes	*Gfap*	AGCGAGCGTGCAGAGATGA	AGGAAGCGGACCTTCTCGAT
Oligodendrocytes	*Cnp*	GCTGCACTGTACAACCAAATTCTG	ACCTCCTGCTGGGCGTATT
Microglia	*Aif1*	CCCCCAGCCAAGAAAGCTAT	GCCCCACCGTGTGACATC

Supplementary References

1. Saunders, A., *et al.* Molecular Diversity and Specializations among the Cells of the Adult Mouse Brain. *Cell* **174**, 1015-1030 (2018).
2. Gong, S., *et al.* A gene expression atlas of the central nervous system based on bacterial artificial chromosomes. *Nature* **425**, 917-925 (2003).
3. Shuen, J.A., Chen, M., Gloss, B. & Calakos, N. Drd1a-tdTomato BAC transgenic mice for simultaneous vizualization of medium spiny neurons in the direct and indirect pathways of the basal ganglia. *J Neurosci* **28**, 2681-2685 (2008).
4. Sawamoto, K., *et al.* Visualization, direct isolation, and transplantation of midbrain dopaminergic neurons. *Proc Natl Acad Sci U S A* **98**, 6423-6428 (2001).
5. Gong, S., *et al.* Targeting Cre recombinase to specific neuron populations with bacterial artificial chromosome constructs. *J Neurosci* **27**, 9817-9823 (2007).
6. Madisen, L., *et al.* A robust and high-throughput Cre reporting and characterization system for the whole mouse brain. *Nat Neurosci* **13**, 133-140 (2010).
7. Sanz, E., *et al.* Cell-type-specific isolation of ribosome-associated mRNA from complex tissues. *Proc Natl Acad Sci U S A* **106**, 13939-13944 (2009).
8. Bello, S.P., *et al.* Cocaine supersensitivity and enhanced motivation for reward in mice lacking dopamine D2 autoreceptors. *Nat Neurosci* **14**, 1033-1038 (2011).
9. Puighermanal, E., *et al.* drd2-cre:ribotag mouse line unravels the possible diversity of dopamine d2 receptor-expressing cells of the dorsal mouse hippocampus. *Hippocampus* **25**, 858-875 (2015).
10. Ceolin, L., *et al.* Cell Type-Specific mRNA Dysregulation in Hippocampal CA1 Pyramidal Neurons of the Fragile X Syndrome Mouse Model. *Front Mol Neurosci* **10**, 340 (2017).
11. Gras, C., *et al.* The vesicular glutamate transporter VGLUT3 synergizes striatal acetylcholine tone. *Nat Neurosci* **11**, 901-911 (2008).
12. Becamel, C., *et al.* Interaction of serotonin 5-hydroxytryptamine type 2C receptosr with PDZ10 of the multi-PDZ domain protein MUPP1. *J Biol Chem* **246**, 12974-12982 (2001).
13. Clark, M;B., *et al.* Mammalian gene PEG10 expresses two reading frames by high efficiency-1 frameshifting in embryonic-associated tissues. *J Biol Chem* **282**, 37359-37369 (2007).
