# Supplementary material for: Functional and molecular heterogeneity of D2R neurons along dorsal ventral axis in the striatum
Source: Nat Commun. 2020 Apr 23;11:1957. doi: 10.1038/s41467-020-15716-9 (PMC7181842; doi:10.1038/s41467-020-15716-9)
Supplement: Supplementary file 3 — Description of Additional Supplementary Files [file 41467_2020_15716_MOESM3_ESM.docx]

Description of Additional Supplementary Files

**Title: Supplementary Video 1.**

**Description:** Representative wild-type control mouse performing the marble burying test (from min 5 to 10).

**Title: Supplementary Video 2.**

**Description:** Representative D2R-cKO mouse performing the marble burying test (from min 5 to 10). Note the decrease in digging behavior compared to wild-type littermates.

**Supplementary Data 1.** Dataset of RNAseq of DS D2R neurons, Acb D2R neurons and Acb Wfs1 neurons

**Supplementary Data 2.** Genes enriched in DS D2R neurons and Acb D2R neurons

**Supplementary Data 3.** Mitochondrial-related and imprinted genes enriched in DS and Acb D2R neurons

**Supplementary Data 4.** Genes enriched in cholinergic interneurons

**Supplementary Data 5.** Gene Ontology analysis of genes of Acb D2R neurons

**Supplementary Data 6.** Gene Ontology analysis of genes of DS D2R neurons

**Supplementary Data 7.** Gene Ontology analysis of genes enriched in DS vs Acb D2R neurons

**Supplementary Data 8.** Genes enriched in Acb Wfs1 neurons

**Supplementary Data 9.** Gene Ontology analysis of genes enriched in Acb Wfs neurons

**Supplementary Data 10.** Acb D2R and Wfs-enriched genes in D2R-Htr7-SPNs, D1R-Pcdh8-SPNs and S1R-Cxcl14-SPNs population
