# Supplementary material for: Functional and molecular heterogeneity of D2R neurons along dorsal ventral axis in the striatum
Source: Nat Commun. 2020 Apr 23;11:1957. doi: 10.1038/s41467-020-15716-9 (PMC7181842; doi:10.1038/s41467-020-15716-9)
Supplement: Supplementary file 16 — Reporting Summary [file 41467_2020_15716_MOESM16_ESM.pdf]

nature research

Corresponding author(s): Emmanuel VALJENT

Last updated by author(s): 2020/02/21

Reporting Summary

Nature Research wishes to improve the reproducibility of the work that we publish. This form provides structure for consistency and transparency in reporting. For further information on Nature Research policies, seeAuthors & Referees and theEditorial Policy Checklist .

Statistics

For all statistical analyses, confirm that the following items are present in the figure legend, table legend, main text, or Methods section.

n/a	Confirmed	
☐	☒	The exact sample size (*n*) for each experimental group/condition, given as a discrete number and unit of measurement
☐	☒	A statement on whether measurements were taken from distinct samples or whether the same sample was measured repeatedly
☐	☒	The statistical test(s) used AND whether they are one- or two-sided<br>*Only common tests should be described solely by name; describe more complex techniques in the Methods section.*
☐	☐	A description of all covariates tested
☐	☒	A description of any assumptions or corrections, such as tests of normality and adjustment for multiple comparisons
☐	☒	A full description of the statistical parameters including central tendency (e.g. means) or other basic estimates (e.g. regression coefficient) AND variation (e.g. standard deviation) or associated estimates of uncertainty (e.g. confidence intervals)
☐	☒	For null hypothesis testing, the test statistic (e.g. *F*, *t*, *r*) with confidence intervals, effect sizes, degrees of freedom and *P* value noted<br>*Give P values as exact values whenever suitable.*
☒	☐	For Bayesian analysis, information on the choice of priors and Markov chain Monte Carlo settings
☒	☐	For hierarchical and complex designs, identification of the appropriate level for tests and full reporting of outcomes
☒	☐	Estimates of effect sizes (e.g. Cohen's *d*, Pearson's *r*), indicating how they were calculated

Our web collection on statistics for biologists contains articles on many of the points above.

Software and code

Policy information about availability of computer code

Data collection	Real Time Analysis software (RTA 1.13.48), CASAVA (FASTQ sequence files)
Data analysis	STAR version 2.5.3a, RSEM version 1.3.0 , R 3.4.2, ‘ggplot2’ R library 3.1.1, DESeq2 version 1.18.1, pheatmap R package 1.0.12, Gprofiler 0.6.1, GraphPad Prism v6.0 software, Noldus Ethovision v11.5.

For manuscripts utilizing custom algorithms or software that are central to the research but not yet described in published literature, software must be made available to editors/reviewers. We strongly encourage code deposition in a community repository (e.g. GitHub). See the Nature Research guidelines for submitting code & software for further information.

Data

Policy information about availability of data

All manuscripts must include a data availability statement. This statement should provide the following information, where applicable:

- Accession codes, unique identifiers, or web links for publicly available datasets
- A list of figures that have associated raw data
- A description of any restrictions on data availability

The data supporting the findings of this study are available within the paper and its Supplementary materials files or available from the corresponding author upon reasonable request.
Sequence data have been deposited in Gene Expression Omnibus, accession code GSE94145. IUPHAR/BPS database (www.guidetopharmacology.org) was used to implement gene classification

Field-specific reporting

Please select the one below that is the best fit for your research. If you are not sure, read the appropriate sections before making your selection.

- [x] Life sciences
- [ ] Behavioural & social sciences
- [ ] Ecological, evolutionary & environmental sciences

For a reference copy of the document with all sections, see nature.com/documents/nr-reporting-summary-flat.pdf

Life sciences study design

All studies must disclose on these points even when the disclosure is negative.

Sample size	For animal experiments, sample size was chosen based on similar previous studies of our group and on the basis of literature documentation of similar well-characterized experiments. We try to use the fewest number of mice to achieve statistical significance without compromising the outcomes. The sample size of all experiments are provided in figure legends.
Data exclusions	In a normal distribution, values that fall outside 2 standard deviations of the means were excluded. Criteria were pre-established. Only a few mice that jumped out of the maze/arena during the task were excluded.
Replication	All attempts to replicate the experiments have been successful (RNAseq and behavioral analysis).<br>Two independent RNAseq experiments have been performed (2016 and 2019).<br>Behavioral experiments have been performed on at least 2 distinct batch of mice.
Randomization	No randomization method was used for RNAseq, WB and immunofluorescence analysis. For behavioral experiments, we always tried to use the same number of male/female mice and wild-type/knock-out mice.
Blinding	All behavioral experiments were done under blind conditions as stated in methods section. Blinding was not applicable and necessary for RNAseq, immunofluorescence and WB experiments.

Reporting for specific materials, systems and methods

We require information from authors about some types of materials, experimental systems and methods used in many studies. Here, indicate whether each material, system or method listed is relevant to your study. If you are not sure if a list item applies to your research, read the appropriate section before selecting a response.

Materials & experimental systems

n/a	Involved in the study	
☐	☒	Antibodies
☒	☐	Eukaryotic cell lines
☒	☐	Palaeontology
☐	☒	Animals and other organisms
☒	☐	Human research participants
☒	☐	Clinical data

Methods

n/a	Involved in the study	
☒	☐	ChIP-seq
☒	☐	Flow cytometry
☒	☐	MRI-based neuroimaging

Antibodies

Antibodies used

Information related to the antibodies used in the study are listed with all details in the resourse table.
HA: Covance (#MMS-101R)
HA: Rockland (#600-401-384)
DARPP-32: Cell Signaling Technology (#2306)
Calretinin: Swant (#7699/3H)
Parvalbumin: Swant (#PV25)
NPY: Abcam (#ab10980)
SOM: Millipore (#AB5494)
Gat1: Millipore (#AB1570)
Gprin1: Proteintech Group (#13771-1-AP)
Hap1: ThermoFischer Scientific (#MA1-46412)
Foxp2: Abcam (#ab16046)
MAP2: Sigma-Aldrich (#M4403)
Sox1: Cell Signaling Technology (#4194)
Nrgn: Santa-Cruz (#sc-50401)
D2R: Frontier Institute (#D2R-Rb-Af960)

ChAT: Millipore (#AB144)
GFP: Life Technologies (#A10262)
NK1R: Sigma-Aldrich (#S8305)
VAChT: Synaptic System (#139103)
VGLUT3: gift from Dr S El Mestikawy (Gras et al., 2008)
TrkA: Millipore (#06-574)
5-HT2c: gift from Dr C Becamel (Becamel et al., 2001)
CB1R: Frontier Institute (#CB1-Rb-Af960)
RFP: MBL (#PM005)
actin: Abcam (#AB6276)
Dlk1: Adipogen (#AG-20A-0057)
Peg10: gift from Dr T Bouschet (Clark et al., 2007)
Nnat: Abcam (#AB27266)
TH: Millipore (#mab318)
GluR2: Millipore (#MAB397)

Validation

The validation of each primary antibody for the species and application is available on the manufacturer's website and in relevant citations for the anti-VGLUT3 (Gras et al., 2008), anti-5HT2c (Becamel et al., 2001) and anti-Peg10 (Clark et al., 2007).

Animals and other organisms

Policy information about studies involving animals; ARRIVE guidelines recommended for reporting animal research

Laboratory animals

The different mouse lines used in the present study are listed in the resource table.
Strain, sex and age of the animals are described in methods section as well as housing and breeding. 8-12 weeks old mice were used in the present study.
- D2-eGFP (Drd2-eGFP), males and females (immunofluorescence)
- D1-eGFP (Drd1a-eGFP) males (immunofluorescence)
- D2-eGFP/D1-tdTomato (Drd2-eGFP/Drd1a-tdTomato) males (immunofluorescence)
- Th-eGFP (Th-eGFP) males (immunofluorescence)
- D2-RiboTag ((Drd2-Cre:RiboTag-loxP/loxP) males (Polysomes IP and RNA seq), males and females (immunofluorescence)
- Wfs1-CreERT2 (Wfs1-Tg3-CreERT2:Cre) males and females (behaviors)
- Wfs1-RiboTag (Wfs1-Tg3-CreERT2:Cre::RiboTag-loxP/loxP) males (Polysomes IP and RNA seq), males and females (immunofluorescence)
- D2R-cKO (Wfs1-Tg3-CreERT2:Cre:Drd2-loxP/loxP) males and females (immunofluorescence, WB and behaviors)
- C57/Bl6 mice males (WB)

Wild animals

No wild animals were used in the study.

Field-collected samples

No field collected samples were used in the study

Ethics oversight

All experiments were in accordance with the guidelines of the French Agriculture and Forestry Ministry for handling animals as stated in methods section.

Note that full information on the approval of the study protocol must also be provided in the manuscript.
